# Supplementary material for: The Adipose Stem Cell as a Novel Metabolic Actor in Adrenocortical Carcinoma Progression: Evidence from an In Vitro Tumor Microenvironment Crosstalk Model
Source: Cancers (Basel). 2019 Dec 4;11(12):1931. doi: 10.3390/cancers11121931 (PMC6966501; doi:10.3390/cancers11121931)
Supplement: Supplementary file 1 [file cancers-11-01931-s001.pdf]

Supplementary Materials

The Adipose Stem Cell as a Novel Metabolic Actor in Adrenocortical Carcinoma Progression: Evidence from an In Vitro Tumor Microenvironment Crosstalk Model

Roberta Armignacco, Giulia Cantini, Giada Poli, Daniele Guasti, Gabriella Nesi, Paolo Romagnoli, Massimo Mannelli, Michaela Luconi

Figure 2B

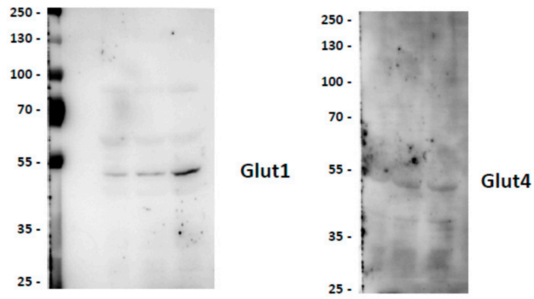

Figure 2D

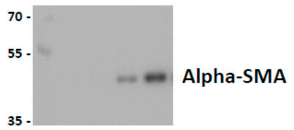

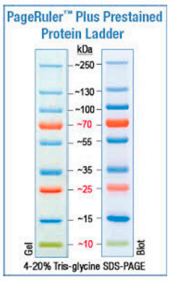

Molecular weight markers: Pageruler Plus Prestained Protein Ladder #26619

Figure 5C

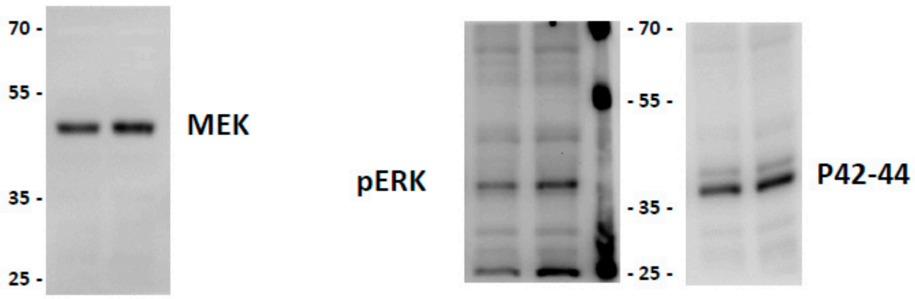

Figure 6D

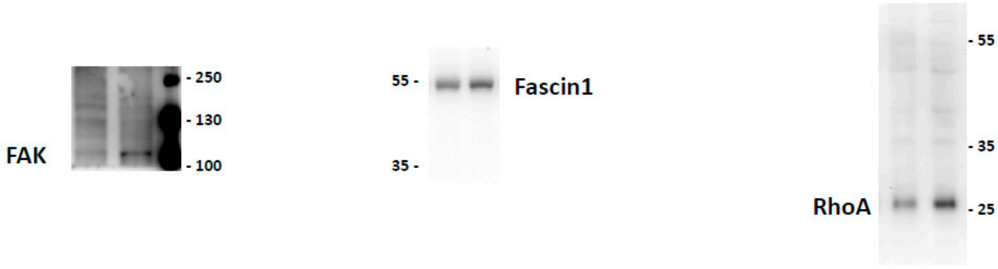

Figure 7C

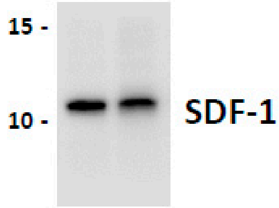

© 2019 by the authors. Licensee MDPI, Basel, Switzerland. This article is an open access article distributed under the terms and conditions of the Creative Commons Attribution (CC BY) license (http://creativecommons.org/licenses/by/4.0/).
